# Early effects of concurrent administration of artesunate-amodiaquine and nifedipine on sperm parameters and sex hormones in guinea pigs: An experimental study

**Published:** 2018-10

**Authors:** Jonah Sydney Aprioku

**Affiliations:** *Department of Experimental Pharmacology and Toxicology, Faculty of Pharmaceutical Sciences, University of Port Harcourt, Nigeria.*

**Keywords:** Artesunate-amodiaquine, Guinea pig, Nifedipine, Sperm motility, Synergy

## Abstract

**Background:**

Many antimalarial agents and calcium channel blockers have been demonstrated to alter male reproductive activity. Increasing prevalence of hypertension, therefore, increases concern of male infertility, and concurrent administration of antihypertensive and antimalarial agents in malaria-prone areas.

**Objective:**

The study evaluates the reproductive effect of co-administration of artesunate (Ats)-amodiaquine (Amod) and nifedipine (Nif) in male guinea pigs.

**Materials and Methods:**

In this experimental study, 24 adult male pigs were divided into four groups (n=6/ each) as one control (given distilled water) and 3 intervention groups (given standard daily dose equivalents of Ats-Amod, Nif or combination of both drugs) for 14 days. Serum levels of testosterone, follicle-stimulating hormone and luteinizing hormone were measured using enzyme-linked immunosorbent assay. Testicular weight was measured and the relative weight (organ-to-body weight ratio) was obtained. Sperm count, motility, and morphology were equally analyzed.

**Results:**

Nif treatment produced no significant effect on the hormone levels (p=0.058) and sperm parameters (p=0.0568) that were measured, whereas Ats-Amod and Ats-Amod+Nif decreased testosterone level (p=0.0482), sperm count and motility (p<0.0001), but failed to cause an alteration in follicle-stimulating hormone, luteinizing hormone and sperm morphology. Percentage of motility reduction by Ats-Amod+Nif was greater (p=0.025) compared to Ats-Amod effect. Relative testicular weight was decreased (p=0.046) by Ats-Amod and Ats-Amod+Nif, but unaffected by Nif.

**Conclusion:**

The result suggests that short-term administration of standard daily dose equivalent of Nif does not alter hormone levels and sperm indices, while Ats-Amod alone or in combination with Nif decreases testosterone, sperm count, and motility. The combination also results in synergistic inhibition of sperm motility.

## Introduction

Malaria is an important public health disease which is caused by the Plasmodium parasite. The Anopheles mosquito transmits the malaria parasite, which breeds well in pools of water in warm, humid climates. The disease proliferates in less developed areas with low awareness and poor health care systems (1). Presently, the disease is endemic in different parts of the world especially Sub-Saharan Africa causing high morbidity and mortality (2). The major available intervention for the disease remains prompt treatment with effective antimalarial drugs, and artemisinin-based combination therapies (ACTs) are recommended as the first line agents (3, 4). They include artesunate-amodiaquine (Ats-Amod), artesunate-sulfadoxine-pyrimethamine, artesunate-mefloquine, artemether-lumefantrine and dihydroartemisinin-piperaquine. 

Hypertension has equally become an important preventable medical condition of public health concern globally, causing high mortality (5, 6). It is a major risk or contributing factor of cardiovascular diseases, strongly influenced by genetic and environmental (lifestyle) factors (7, 8). Similar to the trend of malaria, a large percentage of global cardiovascular disease mortality occurs in low- and middle-income countries (5, 9). The disease has no cure but drugs are available for its management, which requires long-term (life-long) treatment to maintain normal blood pressure. Nifedipine, a calcium channel blocker is among the first-line agents for hypertension therapy (10). It belongs to the 1, 4 dihydropyridine class and decreases peripheral vascular resistance (11).

Antimalarial agents, including ACTs, have been shown to alter male reproductive activity (12, 13). Calcium channel blockers, including Nifedipine have equally been demonstrated to be detrimental to sperm function (14-16). As earlier explained, hypertension is prevalent in economically developed and developing countries, including Sub-Saharan Africa where malaria is endemic (7, 9). Thus, concurrent administration of antihypertensive and antimalarial agents is inevitable. This raises the concern of the toxicological impact of simultaneous administration of calcium channel blockers and antimalarial agents on the male reproductive system in such areas, which has not been studied. Male infertility (male fecundity) commonly arises from a reduction in the quantity and quality of semen (17), and male factor has been identified to account for 40-50% of infertility among couples (18). The guinea pig could be an appropriate animal model for such toxicological studies as it has a comparatively high spermatogenic efficiency (19). Further, the spermatogenic cycle of pigs lasts for about 12 days (based on 4.5 cycles), and the entire spermatogenic process is approximately 55 days, which is not very different in mammals (20).

The objective of this study was to investigate the effect of concurrent treatment with Ats-Amod and Nifedipine daily for 14 days using standard therapeutic dose equivalents of the drugs on sperm parameters (count, motility, and morphology) in Guinea pigs. The effect of the drug treatments on testicular weight, and serum hormone levels of testosterone, follicle-stimulating hormone (FSH) and luteinizing hormone (LH) were equally evaluated.

## Materials and methods

**Drugs**

Ats-Amod (Adams Pharmaceutical Co. Ltd., China), and Nifedipine (Abbot Health Care Pvt Ltd., India.) tablets were purchased from the Pharmacy Unit of the University of Port Harcourt Teaching Hospital, Port Harcourt, Nigeria.

**Animals**

Twenty-four adult male guinea pigs (15 wk old) weighing 500-520 gr were used for this experimental study. They were obtained from the Animal House of the Department of Pharmacology, University of Port Harcourt. They were allowed to acclimate for 14 days before used for the experiment. 

The animals were randomly divided into four groups (n=6/each). Group I served as control and were given distilled water (2 ml/kg). Group II was given Ats-Amod, 4-10 mg/kg/daily, which is equivalent to its standard daily clinical dose for treatment of uncomplicated malaria (21). Group III received Nifedipine, 0.5 mg/kg/daily, which is equivalent to its standard daily clinical dose for control of uncomplicated hypertension (22), and Group IV received a standard daily clinical dose equivalent of Ats-Amod+Nifedipine. 

The tablets were pulverized separately in glass mortar and pestle and administered as a suspension in water by oral gavage. The drug suspension was continuously agitated during administration in order to deliver the drugs homogenously to the animals. The drugs were administered for 14 days and the guinea pigs were killed by cervical dislocation 24 hr after drug administration under deep diethylether anesthesia. Animal body weights were obtained and blood was withdrawn into plain specimen bottles by cardiac puncture and centrifuged. Serum levels of testosterone, FSH and LH were measured by enzyme-linked immunosorbent assay method using a Microplate Reader (Model: Stat Fax-2100; Awareness Technology Inc., Tokyo, Japan). The testes were isolated carefully and weighed and the relative organ weight (organ-to-body weight ratio) was obtained. Sperm was also extracted from the epididymis and sperm motility, count and morphology were analyzed. 

**Sperm analysis**

Sperm analysis was performed using standard laboratory methods as described by Ochei and Kolhatker (23). Briefly, sperm motility was quantified by counting sperms (motile and non-motile) in at least 10 randomly selected fields under the microscope using a magnification of 400× and expressed in percentage. Sperm motility was evaluated immediately after sperm collection. Sperm count was done using the improved Neubauer counting chamber. The chamber was prepared and charged with diluted seminal fluid (1:20) and maintained in a moist chamber for 20 min. Mature spermatozoa were thereafter counted under the microscope using 400× objective. Sperm morphology was analyzed after air-dried sperm smears were stained with two drops of Walls and Ewas and viewed under brightfield optics at 100× objective with oil immersion. Spermatozoa (about 200) were counted and the percentage of abnormal forms was obtained.

**Ethical consideration**

Experimental protocol followed approved guideline by the Animal Ethics Committee of the Port Harcourt, Nigeria (UPH/CHREC/APP/018/2016). The animals were fed with standard rodent chow, maintained under natural conditions, and handled in accordance with international guidelines for care and use of laboratory animals in biomedical research (24).

**Statistical analysis**

Data were analyzed using GraphPad Prism Version 5 software. Data are expressed as mean±standard error of the mean (SEM). The data were analyzed using one-way analysis of variance (ANOVA) followed by Dunnett’s posttest to compare experimental values with control. Values were considered significant at p˂0.05.

## Results

**Effects on sperm parameters**

**Figure 1:**
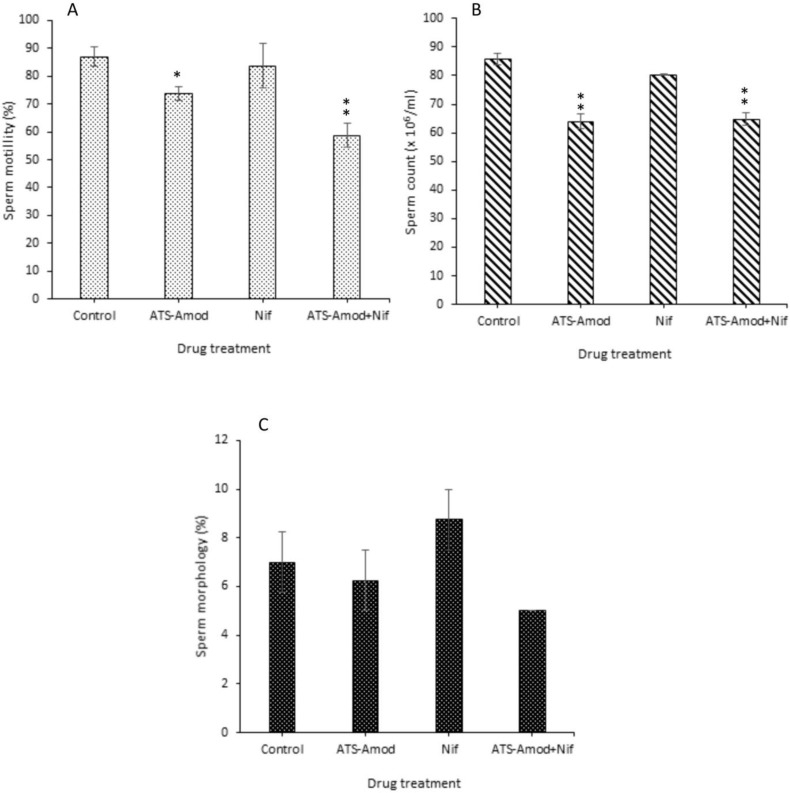
Effects of 14 days oral administration of standard daily dose equivalents of Ats-Amod, Nif, and Ats-Amod+Nif on A) sperm motility, B) sperm count and C) sperm morphology in guinea pigs (n=6/each)

Sperm motility in Ats-Amod treated pigs was reduced (p=0.0008) compared to control, the value obtained was 73.75±2.39% compared to 87.00±3.39% in the control (Figure 1A). There was no significant difference (p=0.0568) between the percentage of motile sperm cells in Nif treated (83.75±8.00%) and control animals (Figure 1A). In pigs treated with Ats-Amod+Nif, the sperm motility obtained (58.75±4.27%) was decreased (p<0.0001) when compared with the control (Figure 1A). Intergroup comparison showed that sperm motility in Ats-Amod+Nif treated was significantly lower (p=0.025) than Ats-Amod or Nif alone treated groups (Figure 1A). 

Furthermore, there was a significant reduction (p<0.0001) of sperm count (64.00±2.61 ×10^12^/ml) in animals that were treated with Ats-Amod compared to the sperm count (85.80±2.08 ×10^12^/ml) that was obtained in the control (Figure 1B). There was no significant difference (p=0.245) between sperm count in Nif treated (80.25±0.25 ×10^12^/ml) and control pigs (Figure 1B). Contrary to Nif result, the sperm count of pigs that received Ats-Amod+Nif (64.75±2.25 ×10^12^/ml) was lower (p<0.0001) compared to control (Figure 1B). 

In addition, comparison of the sperm counts between Ats-Amod and Ats-Amod+Nif treatment groups did not show any significant difference, (p=0.125 Figure 1B). Percentage of morphologically abnormal sperm cells (sperm morphology) in Ats-Amod, Nif, and Ats-Amod+Nif treated pigs were the same compared with the control group, (p=0.405 Figure 1C). 

**Effects on reproductive hormones**

**Figure 2:**
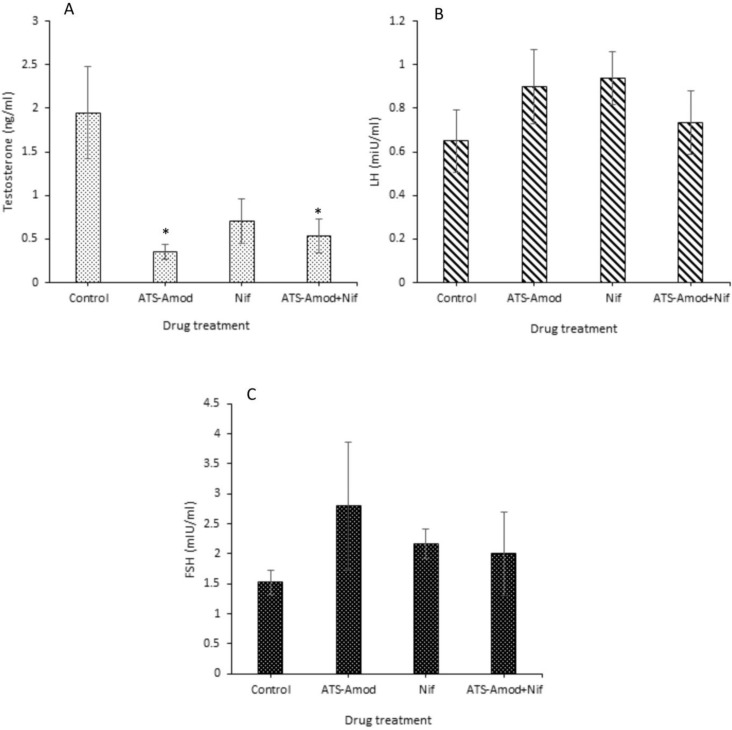
Effects of 14 days oral administration of standard daily dose equivalents of Ats-Amod, Nif, and Ats-Amod+Nif on serum levels of A) testosterone, B) LH and C) FSH in guinea pigs (n=6/each)

In Nif treated pigs, all three hormone (testosterone, LH, and FSH) levels (0.70±0.26 ng/ml, 0.94±0.12 miU/ml, and 2.16±0.25 miU/ml, respectively) were unaffected (p=0.454) when compared with 1.95±0.73 ng/ml, 0.65±1.14 miU/ml, and 1.53±0.21 miU/ml, respectively, that were obtained in control animals (Figures 2A-C). Testosterone level (0.35±0.09 ng/ml) was decreased (p=0.0482), whereas LH (0.90±0.17 miU/ml) and FSH (2.80±1.07 miU/ml) were unaffected in Ats-Amod treated pigs (p=0.5591) when compared to control (Figures 2A-C). This observation was similar in the animals that were given the combination of Ats-Amod and Nif when their hormone levels (0.54±0.19 ng/ml, 0.73±0.15 miU/ml, and 2.00±0.70 miU/ml, respectively) were compared with control (Figures 2A-C).

**Effect of testicular weight**

**Figure 3:**
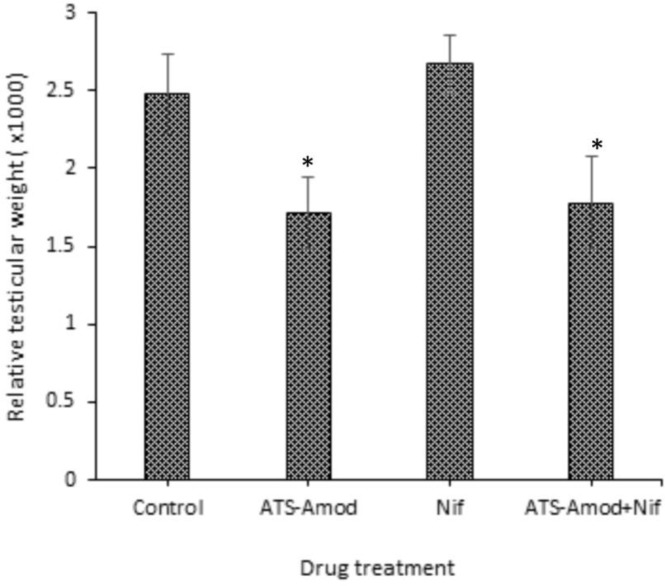
Effects of 14 days oral administration of standard daily dose equivalents of Ats-Amod, Nif, and Ats-Amod+Nif on relative testicular weight in guinea pigs (n=6/each)

The relative testicular weight (organ-to-body weight ratio) of Ats-Amod treated pigs was lower (p=0.0460) compared to control (Figure 3). The average value of testis-to-body weight ratio that was obtained in Ats-Amod+Nif treated pigs was equally lower (p=0.0304) when compared with the control, but there was no change (p=0.0721) in the relative testicular weight of Nif treated pigs (Figure 3).

Values are expressed as mean±SEM. * Significant at p<0.01, ** Significant at p<0.0001.

Ats: artesunate

Amod: amodiaquine

Nif: nifedipine

Values are expressed as mean±SEM. * Significant at p<0.05.

Ats: artesunate

Amod: amodiaquine

Nif: nifedipine

Values are expressed as mean±SEM

* Significant at p<0.05.

Ats: artesunate

Amod: amodiaquine

Nif: nifedipine

## Discussion

This study reports the effects of fourteen days concurrent standard dose treatments with Ats-Amod and Nif on sperm parameters and serum hormone levels in guinea pigs. The former is a commonly prescribed ACT, while the latter is a calcium channel blocker, frequently prescribed for the treatment of hypertension. ACTs are the current first-line drugs for malaria chemotherapy, in view of their high effectiveness in causing parasite clearance and prevention of the emergence of resistance (2, 3). 

Unfortunately, several factors including, drug resistance, weak drug control, and inadequate medical facilities and services in many malaria-endemic regions have encouraged the misuse of these drugs culminating in patients taking the agents for longer durations and at higher doses. This is not without likely drug toxicity. Additionally, the spermatogenic cycle in guinea pigs lasts about 12 days (20) and the sperm transit through the epididymis takes approximately 10 days (19). In this study, the ACT was administered for fourteen days in view of its potential abuse and the spermatogenetic cycle of the guinea pig. The results of the study indicated that daily therapeutic dose treatment with Ats-Amod for fourteen days decreased sperm count and motility, without affecting morphology. This result is in agreement with the findings of previous investigators that performed related studies (12, 13). 

Furthermore, treatment with the calcium blocker, Nif failed to produce an alteration in sperm motility, count or morphology. This observation deviated from previous reports. Iranloye *et al* suggested that oral daily administration of 0.57 mg/kg Nif for 30 days causes reversible deleterious effects on sperm function which is not mediated by a change in testosterone secretion (14). Morakinyo *et al* equally reported that administration of Nif, verapamil, and diltiazem for 30 days appear to have a reversible anti-fertility effect on male rats which does not occur through inhibition of the pituitary-gonadal axis (15). 

In this study, the result with Nif may have been due to the short course of treatment. The implication of this is that Nif is more likely to alter testicular activity after prolonged administration. Interestingly, sperm count and motility were reduced when Ats-Amod+Nif were administered concurrently. But while the extent of sperm count reduction by Ats-Amod+Nif treatment was comparable with that of Ats-Amod treatment alone, the concurrent treatment surprisingly resulted in a higher inhibitory effect on sperm motility. This indicates that Nif combination with Ats-Amod may potentiate the ACT’s negative effect on sperm motility. Calcium ions, among several other functions that determine male fertility, play important role in sperm motility and acrosome reaction (25, 26). 

It is thus logical to suggest that the antagonistic effect on sperm motility by the ACT may involve mechanisms that cause reduction of calcium activity in the testicular milieu, hence the synergistic response with Nif. This finding is important with respect to male infertility, as hypertensive patients on Nif treatment in malaria-endemic regions like the Sub-Sahara are inevitably given ACTs when infected with the malaria parasite. This is novel as the potential interactive effect of artemisinins and antihypertensive drugs on sperm characteristics is not available in existing literatures. Further, relative testicular weight (organ-to-body weight ratio) of Nif administered pigs did not change, which indicates that the drug may not alter testicular function (27, 28). 

This is in agreement with the non-significant spermatogenic effect that was observed in Nif treated animals. Conversely, the relative testicular weight of animals was reduced following ACT treatment, either alone or in combination with Nif. This is suggestive of organ toxicity and correlates positively with the observation of testosterone reduction in the treated animals (27, 28). Testosterone reduction may contribute partly to the negative spermatogenic effect that was induced by the ACT and the combination, as the hormone is essential for spermatogenesis (29). Additionally, FSH and LH levels were not affected throughout, and this suggests that gonadotropic hormone secretion by the anterior pituitary gland may not be affected by the drugs.

## Conclusion

This study has shown that short-term (fourteen days) administration of standard daily dose equivalent of Nif has no effect on sperm indices, while Ats-Amod would cause reductions in testosterone, sperm count and sperm motility. The study also showed that when both drugs are combined, it results in synergistic inhibition of sperm motility. 
